# Neonatal complications and referral practices at birth: insights from a population-based study in the Indian state of Bihar

**DOI:** 10.1136/bmjopen-2024-098408

**Published:** 2025-07-20

**Authors:** G Anil Kumar, Indu Bisht, Md Akbar, S Siva Prasad Dora, Moutushi Majumder, Tanmay Mahapatra, Rakhi Dandona

**Affiliations:** 1 Public Health Foundation of India, New Delhi, India; 2 Piramal Swasthya Management and Research Institute, Hyderabad, India; 3 University of Washington, Seattle, Washington, USA

**Keywords:** EPIDEMIOLOGIC STUDIES, Health Services, Health Surveys, Community child health

## Abstract

**Abstract:**

**Objectives:**

To explore neonatal survival by type of neonatal complications at birth and referral pattern for these complications by place of delivery.

**Setting:**

Bihar, India.

**Participants:**

Women aged 15–49 years who had given live birth between July 2020 and June 2021.

**Primary and secondary measures:**

Prevalence of neonatal complications at birth, referral pattern by complication and neonatal deaths by type of complication.

**Results:**

Data were available for 6767 (81.8%) newborns including 717 neonatal deaths. The prevalence of at least one neonatal complication at birth was reported for 32.9% (95% CI 32.4 to 33.4) newborns, with the most common complications including difficulty in breathing (21.9%), high fever (20.7%), low birth weight (12.5%) and jaundice (13.2%). A total of 578 (26.6%; 95% CI 25.8 to 27.4) neonates with complications at birth were referred to another health provider, predominantly to private sector (68.1%, 93% and 78.7% from public facility, private facility and home). The complications with high referrals included meconium aspiration syndrome (64.1%; 95% CI: 61.1 to 67.1), inability to pass urine (54.7%; 95% CI: 42.1 to 67.2), difficulty in suckling (49.7%; 95% CI: 46.9 to 52.5), cold to touch (48.5%; 95% CI: 43.5 to 53.6), inability to cry (47.2%; 95% CI: 44.2 to 50.1), pneumonia (45.6%; 95% CI: 42.0 to 49.1), difficulty in breathing (44.0%; 95% CI: 42.5 to 45.6) and lethargy (43.5%; 95% CI: 38.4 to 48.6). Referrals were linked to higher neonatal deaths, in particular, among neonates born at home and referred for complications (84.7%; p<0.001) compared with those born in public facilities (59.8%) or private facilities (47.3%) and referred for complications.

**Conclusions:**

With one-third of the neonates reported to have complications at birth and those referred more likely to die, critical gaps in addressing neonatal complications at birth and improvement in the referral services are urgently needed to reduce neonatal mortality.

**STRENGTHS AND LIMITATIONS OF THIS STUDY**

- State-representative sample of all live births in a population over 1 million from an Indian state with high neonatal mortality, covering births in public facilities, private facilities and home births.
- Detailed interviews using standard procedures with the mothers to document the type of neonatal complications at birth and referral pattern for these complications by place of delivery.
- It relies on self-reported data on neonatal complications at birth, which may be subject to recall bias, even though the recall period was short.

## Introduction

 The global neonatal mortality rate (NMR) was estimated at 17.1 per 1000 live births in 2021, with the majority of the neonatal deaths in developing countries.^1^ Neonatal disorders, congenital anomalies and lower respiratory infections were reported as the leading causes of neonatal deaths world-wide.^2 3^ Furthermore, with an estimated 13.4 million babies born preterm,^4^ small size at birth or small-for-gestational-age (SGA) acts as the leading risk factor for over 80% of neonatal deaths.^5^ Many of these neonatal deaths are preventable at large through access to skilled birth attendants and emergency healthcare services during and after the delivery.^6^,^8^ Concerted efforts focused on the type of neonatal complications are required to achieve the Sustainable Development Goal 3 by 2030 to reduce preventable newborn deaths.^9^

The estimated NMR in India for 2021 was 21.2 per 1000 live births.^10^ Several initiatives have been put in place in India with the focus on improving neonatal survival, including setting up of the Newborn Stabilization Units to deliver immediate care to sick and small newborns at subdistrict level.^11^,^17^ An effective referral system is an essential component to enhance the neonatal outcomes,^1618^,^20^ but gaps in the execution of a quality referral system have long been identified in India.^21^,^24^ The lack of knowledge and skills among healthcare professionals to manage major causes of neonatal deaths has also been described as potential factors affecting quality of neonatal care and outcomes.^25^,^27^ In this context, we explore neonatal survival by type of neonatal complications at birth and referral pattern for these complications by place of delivery in a study in the Indian state of Bihar, which is the third most populous state in India with one of the highest rates of neonatal mortality.^28 29^

## Methods

### Study population and design

Detailed methods for ENHANCE 2020 have been published previously.^30 31^ ENHANCE 2020, a cross-sectional study, was designed to determine the change in NMR in Bihar between 2016 and 2020 as compared with the 23.3% decline in NMR documented in Bihar during 2011 to 2016.^32^ Assuming a similar trend, to detect an expected decline in NMR of about 18%, with 85% power and 95% precision, 30 000 live births were estimated as the target sample size for the study, further assuming a 10% refusal rate.

The study population was usual resident women aged 15–49 years, and the inclusion criteria for ENHANCE 2020 was women who reported a birth (live birth and/or stillbirth) between July 2020 and June 2021. By defining the study population as women who had given birth during a set time period, we ensured that all neonates and stillbirths were captured. Defining neonates as the study population would have resulted in a biased sample with under-reporting of neonates who did not survive and of stillborn babies. A usual resident woman was defined as a woman living in the sampled household for at least 6 months prior to the data collection. To ensure a robust estimation of total births in this population, births were documented also for women who had died during or after delivering the baby and those who had migrated out.

We used a multistage sampling design to obtain a representative sample of women with these births from all the 38 districts of Bihar. Each district of Bihar is divided into 5–27 blocks, giving a total of 534 blocks in the state, and we considered 50% of these blocks for the survey. We stratified the 534 blocks as those having only rural population (70.2%) and those with both rural and urban populations (29.8%); and sampled 267 blocks for the survey which included 187 (70%) blocks with only rural population and 80 (30%) blocks with both rural and urban populations. Within these 267 blocks, the secondary sampling units (SSUs) were villages in rural areas and urban frame survey blocks in urban areas as defined by the Census 2011.^33^ Thus, a total of 1340 SSUs were sampled in proportion to the number of villages from each block using systematic random sampling.

### Data collection

Methods relevant to the analysis presented in this paper on live births are described. After the selection and mapping of SSUs, all the households (a household was defined as all the people eating from the same kitchen) of each SSU were enumerated. Trained interviewers captured the information on sociodemography of the household and birth outcomes in usual resident women aged 15–49 years in each household between July 2020 and June 2021 from the head of the household after seeking informed consent. Date of birth and sex of the baby born were documented for each live birth. Following initial documentation, all women with neonatal death and 25% of the women with live births (selected using systematic random sampling in each cluster) were considered eligible for a detailed interview. Informed consent was sought from the sampled women for detailed interview after seeking permission from the head of the household to contact them. The interview captured information on the sociodemography including woman’s age, place of residence, Wealth Index; information related to the focal child including age, sex, birth weight, if the child was currently alive; pregnancy duration for the focal child and the place of delivery were captured. Whether the focal child had any complications at birth was enquired from all the respondents. Those who responded as yes were asked what the complications were using a question with precoded response options. The response options included symptoms (difficulty in breathing, high fever, baby drank dirty water in the womb (meconium aspiration syndrome (MAS)), spasm/convulsion, chest in-drawing, cold to touch, vomiting and difficulty in suckling) and also names of some common conditions (low birth weight, jaundice, pneumonia, diarrhoea) to accommodate for responses given by respondents who may or may not be aware of the clinical diagnosis. All symptoms and conditions indicated by the respondents were captured. The symptoms or clinical diagnosis not in the list were captured in full under ‘others’, which were reviewed by MA and SPD and categorised into meaningful categories (unable to cry, congenital anomalies, infection, measles and lethargy). Furthermore, if the focal child was treated at the place of delivery or was referred for treatment elsewhere for the reported neonatal complications, it was documented, including the type of referral facility referred to.

Data were collected between August 2021 and April 2022. The interview questionnaire was developed in English and then translated into the local language (Hindi) and then again translated back to English to ensure the accuracy and relevant meaning of the questions, without diverging from the intent of the questions. Interviews were conducted by the trained data interviewers using Computer Assisted Personal Interview software in hand-held tablets. A pilot testing of the questionnaire was also carried out to test the logistics and the quality of data collection, and appropriate modifications were made to the questionnaire as required before the start of the survey. A total of 10% of the enumeration data were checked from 50% of the sampled clusters, which translated into 670 clusters. Similarly, a total of 20% of interviews were checked in 50% of the 1340 sampled clusters to ensure data quality.

### Data analysis

We present the findings in two ways—by the type of complications at birth and by neonates with at least one complication at birth. For the type of complications at birth, we considered each episode of complication reported for every neonate as unique to determine the distribution of the reported complication episodes. We present the distribution of the type of reported neonatal complications at birth, overall and by the place of delivery, and also report the prevalence of referral and proportion of neonatal deaths by type of complication.

For findings by neonate, we estimated the prevalence of any reported complications at birth among the live births by select characteristics, overall and disaggregated by place of delivery. We report on the distribution of referral for complications at birth by neonate, the referral pattern by the place of delivery and the care given before referral. The proportion of neonatal deaths overall and by top referred complications is reported among neonates by place of delivery. We report the distribution of overall neonatal deaths and neonatal death disaggregated by age at death (0–2 days, 3–7 days and 8–27 days) and by the single or multiple neonatal complication and its referral status.

As the sample was a multistage stratified cluster sample, sampling weights were calculated based on the sampling probabilities separately for each sampling stage for each study cluster. We calculated the selection probabilities for a cluster from the sample and the birth outcomes; then obtained the overall probability as the product of both of these; sampling weight was estimated by taking the reciprocal of this overall probability. The design weight was then adjusted for household non-response and individual non-response to obtain the final sampling weight. In-transit deliveries were considered as home deliveries. The Wealth Index was calculated using the standard methods used in the National Family Health Survey (NFHS) to calculate the Wealth Index as detailed by the Demographic and Health Survey (DHS) programme for India.^34 35^ We have reported a 95% CI for all estimates as relevant. SAS V.9.4 was used for the analysis.

### Patient and public involvement statement

Patients were not involved in the design, conduct, reporting or dissemination plans of our research.

## Results

A total of 261 124 households were enumerated (91.5% participation) covering a population of 1 260 984 and a total of 30 412 birth outcomes were reported by 29 517 women between July 2020 and June 2021. Of the 30 412 birth outcomes, 29 830 (98.1%) were live births, including 831 (2.8%) neonatal deaths. Of the 8271 eligible women for detailed survey, 6767 (81.8%) women participated, including 717 women with a neonatal death.

### Prevalence of at least one neonatal complication at birth among neonates

Out of 6767 live births, 2007 (32.9%; 95% CI 32.4 to 33.4) neonates were reported to have at least one neonatal complication at birth. Irrespective of the place of delivery, reporting of at least one neonatal complication was significantly higher in neonates with a gestation period of <8 months, whose mother was referred for delivery, those belonging to the highest wealth quartile, in boys and among neonates with a birthweight of <2.5 kg (table 1). The reporting of at least one neonatal complication was significantly higher in newborns delivered at private facilities (44.3%; 95% CI, 43.2 to 45.4) as compared with public facilities (30.4%; 95% CI 29.7 to 31.0) and home deliveries (25.7%; 95% CI 24.7 to 26.6). A similar pattern of prevalence of reporting of at least one neonatal complication was seen by the different places of delivery (table 1).

**Table 1:** Weighted prevalence of reporting of at least one neonatal complication at birth by place of delivery among live births born between 2020–2021 by select characteristics.

Variable	Variable categories	Weighted prevalence of at least one neonatal complication at birth by neonate(95% confidence interval)			
		All live births irrespective of the place of delivery	Public facility delivery	Private facility delivery	Home delivery
Overall		32.9 (32.4 to 33.4)	30.4 (29.7 to 31.0)	44.3 (43.2 to 45.4)	25.7 (24.7 to 26.6)
Urbanicity	Rural	29.9 (29.4 to 30.4)	29.7 (29.0 to 30.4)	40.4 (39.2 to 41.5)	22.8 (21.9 to 23.7)
	Urban	42.6 (41.3 to 43.9)	34.0 (32.4 to 35.7)	48.5 (46.6 to 50.5)	40.2 (36.8 to 43.6)
Wealth index quartile	I (lowest)	31.0 (30.1 to 31.9)	30.5 (29.2 to 31.8)	48.5 (45.5 to 51.4)	25.6 (24.2 to 27.1)
	II	29.7 (28.7 to 30.6)	28.4 (27.3 to 29.6)	41.8 (39.4 to 44.2)	24.5 (22.4 to 26.5)
	III	33.4 (32.4 to 34.3)	29.4 (28.2 to 30.6)	45.5 (43.6 to 47.5)	29.1 (26.8 to 31.4)
	IV (highest)	37.6 (36.5 to 38.7)	34.5 (33.0 to 35.9)	43.3 (41.5 to 45.1)	22.0 (19.9 to 24.2)
Gestation period (months)	<8	88.2 (86.5 to 90.0)	80.6 (76.5 to 84.6)	97.1 (96.2 to 98.1)	81.2 (76.8 to 85.5)
	8	31.4 (30.5 to 32.3)	29.3 (28.2 to 30.5)	43.4 (41.5 to 45.4)	19.8 (18.5 to 21.1)
	More than 8	31.5 (30.9 to 32.1)	29.9 (29.2 to 30.7)	40.9 (39.5 to 42.3)	25.3 (24.1 to 26.6)
Sex of the baby	Boy	37.3 (36.6 to 38.0)	33.4 (32.5 to 34.3)	46.9 (45.3 to 48.4)	33.3 (31.8 to 34.8)
	Girl	28.2 (27.5 to 28.9)	27.2 (26.3 to 28.1)	40.7 (39.2 to 42.3)	19.2 (18.0 to 20.4)
Birth weight	Not weighted	28.8 (27.6 to 30.1)	53.2 (47.3 to 59.0)	43.6 (39.5 to 47.7)	26.8 (25.5 to 28.2)
	Do not know	38.0 (35.5 to 40.6)	33.8 (30.5 to 37.0)	47.8 (43.1 to 52.4)	25.7 (20.0 to 31.3)
	< 2.5 kg	56.3 (54.9 to 57.7)	48.4 (46.8 to 50.1)	75.4 (72.7 to 78.1)	42.9 (38.2 to 47.7)
	>= 2.5 kg	28.5 (27.9 to 29.1)	26.0 (25.3 to 26.7)	36.9 (35.7 to 38.1)	20.5 (19.2 to 21.8)
Referred delivery	No	31.3 (30.8 to 31.8)	30.1 (29.5 to 30.7)	40.9 (39.6 to 42.2)	25.2 (24.3 to 26.2)
	Yes	55.9 (54.1 to 57.7)	47.4 (42.7 to 52.1)	56.9 (54.9 to 58.9)	62.5 (50.8 to 74.2)

### Type of neonatal complications at birth

A total of 3324 complications were reported for 2007 neonates (32.9%), including 963 (47.4%) cases with a single complication and 1044 (52.6%) with multiple complications. Among the 3324 complications, the most commonly reported neonatal complications included difficulty in breathing (21.9%), high fever (20.7%), low birth weight (12.5%) and jaundice (9.8%) as shown in online supplemental table 1. A significantly higher reporting of low birth weight (16.2%; p=0.009), jaundice (13.2%; p=0.009) and MAS (9.6%; p=0.001) was in neonates born in private facilities as compared with those born elsewhere, and high fever (26.3%; p<0.001) and pneumonia (5.3%; p=0.042) were reported significantly more among home births as compared with facility births.

### Referral of neonates for neonatal complications at birth

Among the 2007 neonates with at least one complication, 578 (26.6%; 95% CI 25.8 to 27.4) were referred to another facility or provider for their complications (online supplemental table 2). Overall, referral was significantly higher among neonates from rural areas (p=0.029), with gestation period <8 months (p<0.001), boys (p=0.006) and among neonates whose mother was referred for delivery (p=0.001). The prevalence of referral for complication was the highest in private facility births (36.0%; 95% CI 34.4 to 37.6) as compared with public facility (21.2%; 95% CI 20.1 to 22.2) and home births (22.3%, 95% CI 20.4 to 24.3) but the pattern of referral by select characteristics was similar by place of delivery. Overall, irrespective of the place of delivery, the majority of the referrals was to a private facility—68.1% from public facility, 93.0% from private facility and 78.7% from home (online supplemental table 3). Among the public facility births, referral was higher (though not significant) to a private facility in urban areas (72.4%) and in boys (69.5%), whereas referral to a private facility was significantly higher among girls (95.9%; p=0.022) than boys in home births (online supplemental table 3).

Among the 516 deliveries where neonates were referred, 263 (52.1%) neonates were provided with pretreatment or stabilisation, 82 (13.6%) were put in an incubator, and 175 (31.0%) were provided with oxygen before referral (not mutually exclusive). Pretreatment or stabilisation (63.1%; p=0.014) was reported significantly higher in public facility as compared with private facility and home. Incubator services (27.4%; p<0.001) and oxygen provision (43.4%; p=0.014) were reported significantly higher in public facility as compared with private facility before referral (online supplemental figure 1).

### Referral by type of neonatal complication

Considering the 3324 reported complications irrespective of the place of delivery (table 2), the highest proportion of referrals was for MAS (64.1%; 95% CI: 61.1 to 67.1), followed by inability to pass urine (54.7%; 95% CI: 42.1 to 67.2), difficulty in suckling (49.7%; 95% CI: 46.9 to 52.5), cold to touch (48.5%; 95% CI: 43.5 to 53.6), inability to cry (47.2%; 95% CI: 44.2 to 50.1), pneumonia (45.6%; 95% CI: 42.0 to 49.1), difficulty in breathing (44.0%; 95% CI: 42.5 to 45.6) and lethargy (43.5%; 95% CI: 38.4 to 48.6). Referral was significantly higher for high fever (19.3%; p=0.012), pneumonia (63.1%; p=0.024), chest in-drawing (68.2%; p=0.024) and cold to touch (76.8%; p<0.001) in private facility delivery as compared with public facility delivery and home delivery.

**Table 2:** Weighted prevalence of referral for the type of reported neonatal complications at birth by the place of delivery

Neonatal complication at birth	All live births irrespective of the place of delivery		Live births in public facility		Live births in private facility		Live births at home	
	**Number with complications (N**)	**Prevalence of referral for complication (95% CI**)	**Number with complications (N**)	**Prevalence of referral for complication (95% CI**)	**Number with complications (N**)	**Prevalence of referral for complication (95% CI**)	**Number with complications (N**)	**Prevalence of referral for complication (95% CI**)
Any complication	3324	33.1 (32.4 to 33.8)	1637	28.7 (27.8 to 29.6)	1112	42.9 (41.6 to 44.1)	575	25.2 (23.7 to 26.7)
Difficulty in breathing	740	44.0 (42.5 to 45.6)	343	38.5 (36.3 to 40.8)	280	53.3 (50.8 to 55.7)	117	40.6 (36.6 to 44.7)
High fever	615	11.5 (10.6 to 12.5)	343	11.1 (9.8 to 12.5)	138	19.3 (17.0 to 21.5)	134	4.4 (3.2 to 5.7)
Low birth weight	400	37.2 (34.9 to 39.5)	204	25.9 (23.4 to 28.4)	139	45.6 (41.6 to 49.6)	57	40.5 (34.7 to 46.4)
Jaundice	319	24.7 (22.8 to 26.5)	147	30.3 (26.9 to 33.7)	130	21.0 (18.8 to 23.2)	42	21.4 (16.8 to 26.0)
Difficulty in suckling	193	49.7 (46.9 to 52.5)	94	49.6 (45.3 to 54.0)	55	60.2 (55.4 to 65.0)	44	35.8 (30.2 to 41.4)
Meconium aspiration syndrome*	192	64.1 (61.1 to 67.1)	80	60.2 (55.9 to 64.4)	109	63.8 (59.6 to 68.1)	3	100.0 (100.0 to 100.0)
Unable to cry	170	47.2 (44.2 to 50.1)	86	37.3 (33.2 to 41.3)	61	56.9 (52.1 to 61.8)	23	60.8 (52.4 to 69.3)
Pneumonia	137	45.6 (42.0 to 49.1)	56	49.6 (43.7 to 55.5)	43	63.1 (57.4 to 68.9)	38	21.5 (17.0 to 26.1)
Vomiting	111	8.8 (7.1 to 10.5)	60	6.4 (4.4 to 8.4)	22	15.7 (10.4 to 21.0)	29	7.0 (4.2 to 9.8)
Diarrhoea	85	3.4 (2.3 to 4.5)	47	0.7 (0.3 to 1.2)	22	5.3 (3.0 to 7.5)	16	7.6 (2.8 to 12.5)
Congenital anomalies	73	35.7 (30.9 to 40.4)	36	35.9 (28.9 to 42.9)	23	44.3 (34.4 to 54.1)	14	15.8 (9.7 to 21.9)
Chest in drawing	68	35.7 (31.2 to 40.3)	32	24.2 (19.0 to 29.3)	20	68.2 (57.6 to 78.8)	16	21.1 (14.4 to 27.9)
Cold to touch	63	48.5 (43.5 to 53.6)	26	26.7 (20.9 to 32.5)	19	76.8 (70.3 to 83.4)	18	15.5 (9.9 to 21.0)
Measles	46	19.2 (13.5 to 24.9)	26	9.8 (5.4 to 14.1)	16	27.5 (16.9 to 38.1)	4	17.9 (0.0 to 36.6)
Infection	43	14.5 (11.1 to 18.0)	24	7.5 (4.5 to 10.6)	12	24.4 (15.6 to 33.1)	7	17.1 (4.6 to 29.6)
Lethargic	42	43.5 (38.4 to 48.6)	20	36.8 (28.8 to 44.8)	16	56.0 (46.5 to 65.6)	6	41.6 (23.6 to 59.6)
Unable to pass urine	19	54.7 (42.1 to 67.2)	9	62.9 (46.3 to 79.5)	4	100.0 (100.0 to 100.0)	6	0.0 (0.0 to 0.0)
Spasm/convulsion	8	27.7 (14.9 to 40.5)	4	0.0 (0.0 to 0.0)	3	100.0 (100.0 to 100.0)	1	100.0 (100.0 to 100.0)

All percentages are weighted while the numbers are unweighted.

* Reported as ‘drank dirty water in the womb’.

### Neonatal complications at birth and neonatal deaths

**Figure 1:**
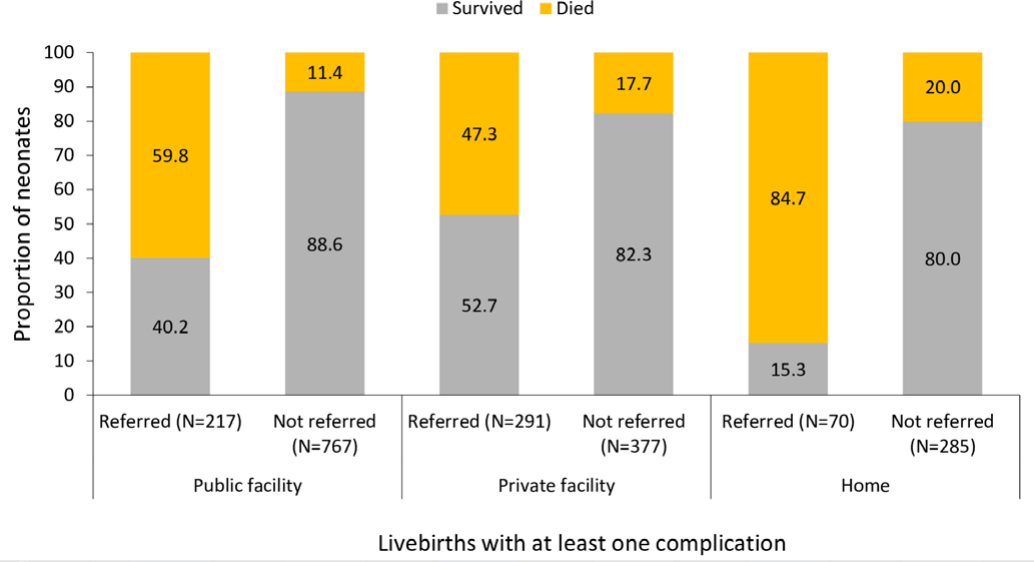
Weighted survival in the neonatal period for neonates with at least one complication at birth by referral and place of delivery.

Irrespective of complications, 715 (10.5%) neonates died during the neonatal period, among whom 451 (61.6%) died during 0–2 days, 147 (18.8%) during 3–7 days and 117 (19.7%) during 8–27 days after birth. The proportion of neonatal deaths was significantly higher among neonates born at home and referred elsewhere for complications (84.7%; p<0.001) compared with those born in public facilities (59.8%) or private facilities (47.3%) and referred for complications. While neonatal death proportions were comparatively lower in the non-referred cases across all settings, the difference in neonatal mortality by the place of delivery remained statistically significant (p=0.019, figure 1).

**Figure 2:**
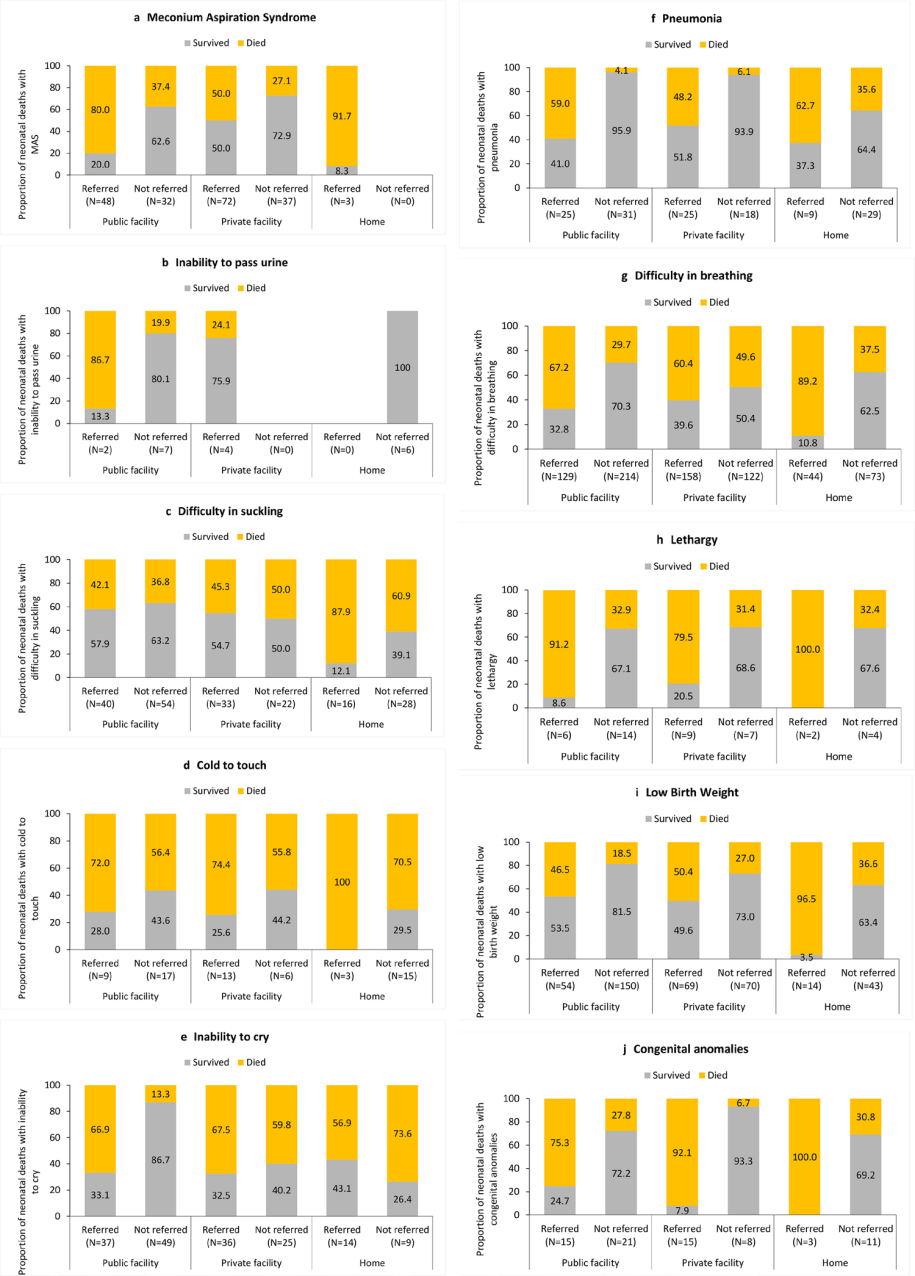
Weighted proportion of survival in the neonatal period for neonates by place of delivery for the top 10 complications at birth based on the prevalence of referral. The complications are not mutually exclusive.

Regardless of the place of delivery, neonatal deaths were more prevalent among the referred neonates with multiple complications (61.7%), followed by those with single complications (43.1%) as compared with those with no reported complications at birth (table 3). The majority of the neonatal deaths were within 0–2 days of birth irrespective of place of delivery or complications. figure 2 shows the survival by referral for the top 10 neonatal complications at birth by the prevalence of referral as identified in table 2 (complications not mutually exclusive). The proportion of neonatal deaths was higher among the referred cases irrespective of the type of complication and place of delivery. On the other hand, the proportion of neonatal deaths was also higher among the non-referred complications in private facilities for most complications as compared with the non-referred neonates for complications in public facilities.

**Table 3:** Weighted distribution of overall neonatal deaths, disaggregated by neonatal death period of 0–2 days, 3–7 days and 8–27 days, among all live births by complication and referral

Place of delivery	Complication at birth	Referred for complication	Number of live births (N)	Neonatal deaths			
				**Total**(**% of N**)	**0–2** days(**% of N**)	**3–7** days(**% of N**)	**8–27** days(**% of N**)
Overall	None	Not applicable	4760	142 (2.7)	81 (1.6)	31 (0.5)	30 (0.6)
	Single	Yes	167	65 (43.1)	42 (27.9)	14 (9.3)	9 (5.8)
		No	796	90 (8.0)	73 (6.3)	7 (0.9)	10 (0.7)
	Multiple	Yes	411	247 (61.7)	141 (35.4)	67 (13.7)	39 (12.6)
		No	633	171 (24.1)	114 (15.2)	28 (3.5)	29 (5.4)
Public facility	None	Not applicable	2595	66 (2.2)	28 (0.9)	18 (0.5)	20 (0.8)
	Single	Yes	55	21 (49.7)	13 (25.9)	4 (14.4)	4 (9.5)
		No	423	31 (6.4)	26 (4.5)	2 (1.0)	3 (0.8)
	Multiple	Yes	162	106 (62.6)	56 (32.5)	29 (14.3)	21 (15.8)
		No	344	68 (18.3)	45 (12.9)	11 (2.0)	12 (3.3)
Private facility	None	Not applicable	960	28 (2.6)	20 (1.7)	4 (0.6)	4 (0.3)
	Single	Yes	95	29 (22.4)	21 (18.3)	6 (2.5)	2 (1.6)
		No	215	33 (7.9)	28 (7.0)	2 (0.5)	3 (0.3)
	Multiple	Yes	196	98 (54.1)	59 (28.6)	29 (14.5)	10 (11.1)
		No	162	53 (31.2)	32 (16.0)	10 (5.4)	11 (9.8)
Home	None	Not applicable	1205	48 (3.9)	33 (2.8)	9 (0.5)	6 (0.7)
	Single	Yes	17	15 (87.3)	8 (60.5)	4 (17.3)	3 (9.6)
		No	158	26 (13.0)	19 (10.5)	3 (1.5)	4 (1.0)
	Multiple	Yes	53	43 (83.8)	26 (64.8)	9 (9.5)	8 (9.5)
		No	127	50 (27.5)	37 (19.3)	7 (4.3)	6 (3.9)

All percentages are weighted while the numbers are unweighted.

## Discussion

One-third of all newborns were reported to have complications at birth, of whom one-fourth were referred to another provider for the complication management in this population, with private sector providers preferred for referral. The neonatal survival in referred newborns with complications was lower than those who were not referred irrespective of the place of delivery. These findings underscore the critical need to strengthen the management of neonatal complications at birth to reduce neonatal mortality in the state.

At least one neonatal complication at birth was reported for one in three newborns in this study, with half of them reported to have more than one neonatal complication. Extrapolating this finding to the estimated 2 560 000 live births in Bihar annually,^1^ 84 750 newborns in the state would likely have at least one neonatal complication at birth. Difficulty in breathing, high fever, low birth weight and jaundice were the most commonly reported neonatal complications at birth with some variation reported by the place of delivery. With the high proportion of facility births in this population, there is a great opportunity for providing essential newborn care and identifying and managing high-risk newborns. Significant investments have been made in India to strengthen care around the time of birth and the first week of life, including expanding services for small and sick newborns and setting up Special Newborn Care Units (SNCUs) in almost all districts of India.^11^,^1315^ Several initiatives have been undertaken in Bihar to improve neonatal survival by addressing skills, capacity and infrastructure-related challenges in the public sector facilities, including for addressing neonatal complications.^36 37^ Logistical, cultural, monitoring and supervision and skill retention barriers have also been identified as challenges to address neonatal complications appropriately and in a timely manner.^38^,^40^ This population-level assessment highlights the magnitude of these challenges, as a significantly higher proportion of neonatal deaths occurred in those newborns with complications than without.

The increased risk of neonatal complications at birth with preterm birth, low birth weight and sex of the neonate found in our study has also been reported previously.^441^,^44^ Gestational age at delivery is considered among major determinants of neonatal survival and morbidity, and low birth weight neonates are more prone to complications,^45 46^ as also observed in our study.^45^,^47^

The significantly higher proportion of neonatal deaths among the referred newborns with neonatal complications in this population is concerning. The majority of these deaths were in 0–2 days, with the most on day 0, highlighting issues with quality, timeliness and appropriateness of referral and of the critical components of management of the neonate.^24 48 49^ Reported complications of MAS, inability to pass urine, difficulty in suckling, cold to touch, inability to cry, pneumonia and difficulty in breathing and lethargy were the complications that resulted in most referrals in this population, which are mostly linked to respiratory distress or prematurity.^50^,^54^ Furthermore, the distribution of referral pattern by the type of complications and by the place of delivery in this study generates important insights both for designing targeted programmes to strengthen the skills and capacity by the place of delivery and to improve on the referral system to reduce neonatal mortality. The majority of the referrals from the public sector facilities was made to the private facilities, highlighting the continued challenges that need to be addressed to strengthen the public sector facilities in the state.^38^,^40^ This brings forward the need for further deep dives to explore how an adequate and comprehensive approach to neonatal care can be provided, addressing not only the infrastructural challenges but also trained workforce and organised referral protocols and systems to ensure timely and effective care for small and sick neonates.

Interestingly, the proportion of referrals for neonatal complications was higher from the private sector than public sector facilities, and these referrals were also predominately to another private sector facility. Also, the proportion of neonatal deaths was higher among the non-referred complications in private facilities for most complications as compared with the non-referred neonates for complications in public facilities. These findings need to be interpreted with caution. The private sector health facilities in Bihar are not a homogenous group and range from tertiary care hospitals to nursing homes with varied levels of infrastructure, capacity and skills.^55^ Newborn survival in India is known to be influenced by the place of delivery, with the private sector performing poorly for early neonatal deaths in comparison to the public sector, including in Bihar.^56^ It is also important to interpret the higher neonatal mortality in private sector facilities within the context of referral for delivery in this population, where most deliveries that were referred were from the public sector to the private sector facilities. It is well known that the private sector in India provides most of the emergency obstetric care and also serves as a referral facility for the public sector for complicated deliveries.^57 58^ Better engagement with the private sector in improving and sustaining quality of care for mothers and newborns is desired under the India Newborn Action Plan.^14^ In view of these findings, appropriate engagement with the private sector in the state is much needed to improve neonatal survival.

Prereferral care was limited in this population irrespective of the type of facility, highlighting the gaps in timely and adequate pre-referral management. Inadequate prereferral care for neonatal complications contributes to poor conditions at arrival with subsequent poor outcome,^19^ whereas neonatal outcomes improve when prereferral care such as administration of intravenous fluid, provision of warmth and/or oxygen administration is provided before referral.^59^ This calls for improved resources and protocols across all facility types to ensure better neonatal outcomes within the referral system. The components include prereferral management, information about availability of facilities for specifically required advanced care, reaching those health facilities without delay with continued care during transit and immediate provision of required treatment including care at SNCU in the referred centre.^19 22 24 60 61^

Several approaches are available towards appropriate care of sick neonates, such as an emergency call and ambulance dispatch centre; mobile application for initiating referral, tracking and deployment of ambulances can improve coordination and efficiency in emergency case referral and transport and an e-referral system to strengthen the health system gaps by streamlining the referral process for timely management of cases.^49 62^ An e-referral system called ‘Jiyyo Innovations e-Referral System’ in India has shown its impact on streamlining the referral process and improved outcomes, by addressing the communication gap between referring and referred facility and by ensuring the timely provision of requisite information about every referral.^61^

The strength of this study lies in the state-wide representative sample of live births capturing the prevalence of neonatal complications, referral patterns and neonatal survival. This large-scale study allows for a detailed understanding of neonatal complications by place of delivery in a low-resource region, which can be generalised for other similar settings as well. Some limitations are to be considered as well. Analysis relies on self-reported data regarding neonatal complications at birth, which may be subject to recall bias, though we only dealt with the recent incidences and the recall period was short. Additionally, the accuracy of the reported complications could be influenced by the respondents’ understanding of the medical condition of the neonates or the severity of the conditions and may not always accurately align with clinical diagnoses. The gestational age was captured in months instead of weeks as the pregnancy length in India is reported in months. The last menstrual period forms the basis for most gestational age estimates and is considered a reliable estimate for measuring gestational age in both developing and developed country settings.^63 64^

### Conclusion

With neonatal complications being reported for one-third of newborns and a significant number of neonatal deaths among those who are referred, the findings call for urgent strengthening of the referral process, ensuring timely access to skilled care and improving the prereferral care to reduce neonatal mortality both in the public and private sector facilities.

## Supplementary material

10.1136/bmjopen-2024-098408 online supplemental file 1

## Data Availability

All data relevant to the study are included in the article or uploaded as supplementary information.
