# Supplementary material for: Neonatal complications and referral practices at birth: insights from a population-based study in the Indian state of Bihar
Source: BMJ Open. 2025 Jul 20;15(7):e098408. doi: 10.1136/bmjopen-2024-098408 (PMC12278138; doi:10.1136/bmjopen-2024-098408)
Supplement: online supplemental file 1 [file bmjopen-15-7-s001.docx]

12 June 2025

**Supplementary material**

**Neonatal complications and referral practices at birth:**

**Insights from a population-based study in the Indian state of Bihar**

Correspondence to: Prof. Rakhi Dandona; [rakhi.dandona@phfi.org](mailto:rakhi.dandona@phfi.org)

**Supplemental table 1. Distribution** **of neonatal complications at birth in Bihar, overall and by the places of delivery.**

| Neonatal complication at birth | Overall | Public facility | Private facility | Home births | P values from Chi square test for significance |
| --- | --- | --- | --- | --- | --- |
|  | **Number of complications (% to N)** | **Number of complications (% to N)** | **Number of complications (% to N)** | **Number of complications (% to N)** |  |
|  | **N=3,324** | **N=1,637** | **N=1,112** | **N=575** |  |
| Difficulty in breathing | 740 (21.9) | 343 (22.2) | 280 (21.3) | 117 (22.4) | 0.911 |
| High fever | 615 (20.7) | 343 (23.9) | 138 (13.7) | 134 (26.3) | <.001 |
| Low birth weight | 400 (12.5) | 204 (10.6) | 139 (16.2) | 57 (10.2) | 0.009 |
| Jaundice | 319 (9.8) | 147 (8.2) | 130 (13.2) | 42 (7.4) | 0.009 |
| Difficulty in suckling | 193 (4.8) | 94 (5) | 55 (4) | 44 (5.9) | 0.319 |
| Meconium Aspiration Syndrome* | 192 (6) | 80 (5) | 109 (9.6) | 3 (1.5) | 0.001 |
| Unable to cry | 170 (4.3) | 86 (4.8) | 61 (4.4) | 23 (2.6) | 0.193 |
| Pneumonia | 137 (3.7) | 56 (4.1) | 43 (2.5) | 38 (5.3) | 0.042 |
| Vomiting | 111 (3.3) | 60 (3.5) | 22 (2.2) | 29 (4.8) | 0.068 |
| Diarrhoea | 85 (2.1) | 47 (2.4) | 22 (1.7) | 16 (2.4) | 0.544 |
| Congenital anomalies | 73 (2.3) | 36 (2.1) | 23 (2.7) | 14 (2.3) | 0.824 |
| Chest in drawing | 68 (1.9) | 32 (1.9) | 20 (1.5) | 16 (2.6) | 0.473 |
| Cold to touch | 63 (1.7) | 26 (1) | 19 (2.3) | 18 (2.2) | 0.052 |
| Measles | 46 (1.8) | 26 (1.8) | 16 (2.7) | 4 (0.2) | 0.010 |
| Infection | 43 (1.2) | 24 (1.3) | 12 (0.9) | 7 (1.7) | 0.547 |
| Lethargic | 42 (1.2) | 20 (1.3) | 16 (1) | 6 (1.3) | 0.826 |
| Unable to pass urine | 19 (0.5) | 9 (0.8) | 4 (0.1) | 6 (0.6) | 0.078 |
| Spasm/convulsion | 8 (0.2) | 4 (0.3) | 3 (0.1) | 1 (0.1) | 0.306 |

*reported by the respondent as “drank dirty water in the womb”.

All percentages are weighted while the numbers are unweighted.

**Supplemental table 2. Referral pattern for neonates with at least one reported neonatal complication at birth in Bihar.**

| Variables | Variable categories | All livebirths | |  | Public facility livebirths | |  | Private facility livebirths | |  | Home livebirths | | |
| --- | --- | --- | --- | --- | --- | --- | --- | --- | --- | --- | --- | --- | --- |
|  |  | **Number of neonates with neonatal complications** | **Number of neonates referred** | **Chi-Square Test p value** | **Number of neonates with neonatal complications** | **Number of neonates referred** | **Chi-Square Test p value** | **Number of neonates with neonatal complications** | **Number of neonates referred** | **Chi-Square Test p value** | **Number of neonates with neonatal complications** | **Number of neonates referred** | **Chi-Square Test p value** |
|  |  | **N** | **N (%)** |  | **N** | **N (%)** |  | **N** | **N (%)** |  | **N** | **N (%)** |  |
| Overall |  | 2,007 | 578 (26.6) |  | 984 | 217 (21.2) |  | 668 | 291 (36.0) |  | 355 | 70 (22.3) |  |
| Urbanicity | Rural | 1,608 | 473 (27.5) | 0.029 | 833 | 191 (21.7) | 0.013 | 473 | 220 (44.0) | 0.004 | 302 | 62 (24.3) | 0.041 |
|  | Urban | 399 | 105 (19.7) |  | 151 | 26 (10.6) |  | 195 | 71 (26.7) |  | 53 | 8 (8.7) |  |
| Wealth index quartile | I (Lowest) | 477 | 125 (23.5) | 0.578 | 242 | 57 (20.6) | 0.901 | 101 | 46 (43.2) | 0.324 | 134 | 22 (16.8) | 0.363 |
|  | II | 478 | 129 (22.6) |  | 276 | 59 (18.0) |  | 113 | 52 (37.2) |  | 89 | 18 (19.3) |  |
|  | III | 528 | 164 (27.2) |  | 258 | 59 (21.4) |  | 182 | 87 (39.1) |  | 88 | 18 (21.4) |  |
|  | IV (Highest) | 522 | 160 (26.6) |  | 207 | 42 (19.0) |  | 271 | 106 (30.3) |  | 44 | 12 (36.4) |  |
| Gestation period (months) | <8 | 116 | 54 (55.1) | <.001 | 34 | 12 (52.0) | 0.001 | 47 | 28 (58.8) | 0.048 | 35 | 14 (51.2) | 0.001 |
|  | 8 | 602 | 172 (26.6) |  | 274 | 62 (23.6) |  | 230 | 91 (33.4) |  | 98 | 19 (17.9) |  |
|  | >8 | 1,289 | 352 (21.4) |  | 676 | 143 (16.9) |  | 391 | 172 (32.0) |  | 222 | 37 (16.9) |  |
| Sex of the baby | Boy | 1,169 | 389 (28.3) | 0.006 | 567 | 147 (23.7) | 0.007 | 400 | 194 (36.0) | 0.649 | 202 | 48 (24.8) | 0.096 |
|  | Girl | 838 | 189 (20.6) |  | 417 | 70 (14.4) |  | 268 | 97 (33.4) |  | 153 | 22 (14.4) |  |
| Referred delivery | No | 1,752 | 466 (23.6) | 0.001 | 945 | 205 (19.5) | 0.149 | 461 | 196 (34.4) | 0.706 | 346 | 65 (19.1) | <0.001 |
|  | Yes | 255 | 112 (37.9) |  | 39 | 12 (32.7) |  | 207 | 95 (36.6) |  | 9 | 5 (74.3) |  |

All percentages are weighted while the numbers are unweighted.

**Supplemental table 3. Referral pattern across places of delivery for neonates with complications at birth, stratified by select characteristics.**

|  |  | Public facility livebirths | | | | Private facility livebirths | | | | Home livebirths | | | |
| --- | --- | --- | --- | --- | --- | --- | --- | --- | --- | --- | --- | --- | --- |
| Variables | **Variable categories** | **Number of neonates referred** | **Referred to public facility** | **Referred to private facility** | **Chi-square test P value** | **Number of neonates referred** | **Referred to public facility** | **Referred to private facility** | **Chi-square test P value** | **Number of neonates referred** | **Referred to public facility** | **Referred to private facility** | **Chi-square test P value** |
|  |  | N | N (%) | N (%) |  | N | N (%) | N (%) |  | N | N (%) | N (%) |  |
| Overall |  | 217 | 71 (31.9) | 146 (68.1) |  | 291 | 28 (7.0) | 263 (93.0) |  | 70 | 10 (21.3) | 60 (78.7) |  |
| Urbanicity | Rural | 191 | 64 (32.4) | 127 (67.6) | 0.713 | 71 | 10 (4.8) | 61 (95.2) | 0.189 | 8 | 2 (5.8) | 6 (94.2) | 0.086 |
|  | Urban | 26 | 7 (27.6) | 19 (72.4) |  | 220 | 18 (8.5) | 202 (91.5) |  | 62 | 8 (23.1) | 54 (76.9) |  |
| Wealth index quartile | I (Lowest) | 57 | 22 (32.7) | 35 (67.3) | 0.989 | 46 | 6 (8.3) | 40 (91.7) | 0.846 | 22 | 4 (30.8) | 18 (69.2) | 0.440 |
|  | II | 59 | 23 (32.6) | 36 (67.4) |  | 52 | 5 (9.9) | 47 (90.1) |  | 18 | 1 (4.6) | 17 (95.4) |  |
|  | III | 59 | 14 (29.4) | 45 (70.6) |  | 87 | 7 (6.7) | 80 (93.3) |  | 18 | 3 (29.5) | 15 (70.5) |  |
|  | IV (Highest) | 42 | 12 (33.5) | 30 (66.5) |  | 106 | 10 (5.7) | 96 (94.3) |  | 12 | 2 (13.6) | 10 (86.4) |  |
| Sex of the baby | Boy | 147 | 44 (30.5) | 103 (69.5) | 0.633 | 194 | 17 (7.1) | 177 (92.9) | 0.971 | 48 | 9 (28.2) | 39 (71.8) | 0.022 |
|  | Girl | 70 | 27 (35.1) | 43 (64.9) |  | 97 | 11 (6.9) | 86 (93.1) |  | 22 | 1 (4.1) | 21 (95.9) |  |

All percentages are weighted while the numbers are unweighted.

**Supplemental figure 1. Weighted distribution of the care given to neonates before referral by place of delivery. (Data missing for 62 cases. Bar represents 95% confidence interval.)**
