# Supplementary material for: Neonatal complications and referral practices at birth: insights from a population-based study in the Indian state of Bihar
Source: BMJ Open. 2025 Jul 20;15(7):e098408. doi: 10.1136/bmjopen-2024-098408 (PMC12278138; doi:10.1136/bmjopen-2024-098408)
Supplement: Supplementary file 2 [file bmjopen-2024-098408.reviewer_comments.pdf]

PEER REVIEW HISTORY

BMJ Open publishes all reviews undertaken for accepted manuscripts. Reviewers are asked to complete a checklist review form and are provided with free text boxes to elaborate on their assessment. These free text comments are reproduced below.

ARTICLE DETAILS

Title (Provisional)

Neonatal complications and referral practices at birth: Insights from a population-based study in the Indian state of Bihar

Authors

Kumar, G Anil; Bisht, Indu; Akbar, Md.; Dora, Siva Prasad; Majumder, Moutushi; Mahapatra, Tanmay; Dandona, Rakhi

---

VERSION 1 - REVIEW

**Reviewer**	**1**
**Name**	**Mwita, Stanley**
**Affiliation**	**Catholic University of Health and Allied Sciences Bugando**
**Date**	**09-May-2025**
**COI**	**None**

---

1. Abstract: Revise the conclusion. The conclusion includes a recommendation but lacks a statement that summarizes the study's findings.

2. Line 42: In this background, I think the author meant to write "In this study."

3. Methods: Line 19 and Abstract line 17. "i) All births that occurred between July 2020 and June 2021 among usual resident women aged 15–49 years were considered eligible for this study. ii) Women aged 15-49 years who had given livebirth between July 2020 to June 2021." The study population is not clear, as the title and results are all about Neonatal complications. Why were neonates not considered as the study population?

4. Methods: Give a detailed description of the study population, as well as the inclusion and exclusion criteria that were applied while recruiting study participants.

5. Discussion: Page 16, lines 8–41. Compare your findings with the results of previous studies.

6. Discussion: In general, the majority of the results discussed in this section were not compared with the findings of previous studies.

Reviewer 2

Name Tibaijuka, Leevan

Affiliation Mbarara University of Science and Technology, Department of Obstetrics and Gynecology

Date 23-May-2025

COI None

This a great paper, tackling a very important aspect of neonatal outcomes and citing inadequacies in the referral processes as a significant contributor to neonatal morbidity and mortality. I however note some areas that could benefit from improvements, below;

1. The abstract lacks an the objective of the study, it states largely a short background but does not explicitly state the study objective. Kindly improve!

2. The study also does not speak to the study design. Was this a cross sectional survey? Kindly make it clear to the reader.

3. The methods are otherwise clearly stated, referencing the ENHANCE 2020 study. The study methodology was rich involving quite a wide coverage of household in the 38 districts of Bihar, this strengthens the generalizability of the findings to a similar setting.

4. The important variables relating to the neonatal complications were not properly collected OR perhaps defined, they seem to mix the clinical diagnoses with symptoms of the conditions being referred to. For example a neonate with meconium aspiration syndrome OR pneumonia may present with difficulty in breathing, chest-indrawing, OR high fever, cold to touch (neonatal sepsis?) etc. Hence stating difficulty in breathing as complications and later pneumonia is quite confusing to the reader on what the authors defined as neonatal complications before data collection. Please provide some clarity on this.

5. Additionally, defining the common complications given the reported symptoms would have created more clarity to this work.

6. a) The analysis could benefit from a review incorporating the above suggestions in regards to handling of the outcome of neonatal complications.

b) Also, inferential statistics beyond description could have provided additional insights on whether the referral processed was indeed associated with the neonatal complications, adjusting for potential confounders.

7. In the current state, the results are largely descriptive and can be improved to give additional insights on the inferential statistics

8.The discussion and conclusions are rich and come from the results presented. But could be enriched with information from additional analyses.

VERSION 1 - AUTHOR RESPONSE

Reviewer: 1

1. Abstract: Revise the conclusion. The conclusion includes a recommendation but lacks a statement that summarizes the study's findings.

   Thank you. We have now revised the conclusion as suggested.

2. Line 42: In this background, I think the author meant to write "In this study."

   Thank you. We have revised this sentence (lines 76-79), to make it clearer.

3. Methods: Line 19 and Abstract line 17. "i) All births that occurred between July 2020 and June 2021 among usual resident women aged 15–49 years were considered eligible for this study. ii) Women aged 15-49 years who had given livebirth between July 2020 to June 2021." The study population is not clear, as the title and results are all about Neonatal complications. Why were neonates not considered as the study population?

   Neonates are not considered the study population as the study documented neonates who were alive, neonates who died, and stillbirths. Defining neonates as the study population would have resulted in a biased sample with under-reporting of neonates who did not survive and of stillborn babies. By defining the study population as women who gave had given birth during a set time period, we ensured that all neonates and stillbirths were captured.

   This paper reports only on livebirths as the primary analysis on neonates who were born alive. We have now added this clarity in line 105.

4. Methods: Give a detailed description of the study population, as well as the inclusion and exclusion criteria that were applied while recruiting study participants.

   The inclusion criteria are already stated in lines 89-94. However, we have now modified the language to make distinction between the study population (and inclusion criteria) for the overall study (ENHANCE 2020) and that for the population that is relevant for this paper (lines 105 and 109-112).

5. Discussion: Page 16, lines 8–41. Compare your findings with the results of previous studies.

   We have compared our study findings with other studies and policy actions as relevant throughout the discussion. A total of 37 references are cited in discussion for such a comparison (references 4, 11-17, 19, 24, and 36-62).

6. Discussion: In general, the majority of the results discussed in this section were not compared with the findings of previous studies.

   As noted above.

Reviewer: 2

This a great paper, tackling a very important aspect of neonatal outcomes and citing inadequacies in the referral processes as a significant contributor to neonatal morbidity and mortality. I however note some areas that could benefit from improvements, below.

Thank you.

1. The abstract lacks the objective of the study, it states largely a short background but does not explicitly state the study objective. Kindly improve!

   Thank you, this was an oversight. We have now added the objectives.

2. The study also does not speak to the study design. Was this a cross sectional survey? Kindly make it clear to the reader.

   It was a cross-sectional study. This is now added in lines 83-84.

3. The methods are otherwise clearly stated, referencing the ENHANCE 2020 study. The study methodology was rich involving quite a wide coverage of household in the 38 districts of Bihar, this strengthens the generalizability of the findings to a similar setting.

   Thank you.

   The important variables relating to the neonatal complications were not properly collected OR perhaps defined, they seem to mix the clinical diagnoses with symptoms of the conditions being referred to. For example a neonate with meconium aspiration syndrome OR pneumonia may present with difficulty in breathing, chest-indrawing, OR high fever, cold to touch (neonatal sepsis?) etc. Hence stating difficulty in breathing as complications and later pneumonia is quite confusing to the reader on what the authors defined as neonatal complications before data collection. Please provide some clarity on this. Additionally, defining the common complications given the reported symptoms would have created more clarity to this work.

   Thank you. We have added more details in lines 115-125 to clarify this. The list of complications was pre-defined and included both symptoms and also names of some common conditions to accommodate for responses given by respondents who may or may not be aware of the clinical diagnosis. In other words, we recorded symptoms or clinical diagnosis as reported by the respondent. Symptoms or clinical diagnosis that were not in the list were documented in full and categorized later.

   This type of data collection reflects the reality of communities in these settings struggling to reduce neonatal mortality. It is not possible to define complications clinically because this is not clinical documentation. Unfortunately, as the reviewer may be aware, poor documentation in medical records is one of the major barriers to effectively understand the clinical aspects to reduce neonatal mortality in less developed settings. This is why we continue to rely on community surveys such as the Demographic Health Survey and surveys such as ours. This is also indicated as limitation of this study in discussion (lines 343-345).

4. a) The analysis could benefit from a review incorporating the above suggestions in regards to handling of the outcome of neonatal complications.
   b) Also, inferential statistics beyond description could have provided additional insights on

whether the referral processed was indeed associated with the neonatal complications, adjusting for potential confounders.

Thank you. As explained above, the suggested analysis is not possible. The study was not designed to explore if the referral processed was indeed associated with the neonatal complications.

5. In the current state, the results are largely descriptive and can be improved to give additional insights on the inferential statistics.

   Thank you. As noted above, we have analysed what is feasible from the available data. We do wish that clinical data availability and adequacy improves in this setting for such data to have more clinical inferences.

6. The discussion and conclusions are rich and come from the results presented. But could be enriched with information from additional analyses.

   Thank you. We are limited by the type of data as indicated above.
